# Single‐cell transcriptomes of murine bone marrow stromal cells reveal niche‐associated heterogeneity

**DOI:** 10.1002/eji.201848053

**Published:** 2019-06-07

**Authors:** Richard K. Addo, Frederik Heinrich, Gitta Anne Heinz, Daniel Schulz, Özen Sercan‐Alp, Katrin Lehmann, Cam Loan Tran, Markus Bardua, Mareen Matz, Max Löhning, Anja E. Hauser, Andrey Kruglov, Hyun‐Dong Chang, Pawel Durek, Andreas Radbruch, Mir‐Farzin Mashreghi

**Affiliations:** ^1^ Deutsches Rheuma‐Forschungszentrum (DRFZ) an Institute of the Leibniz Association Berlin Germany; ^2^ Sanofi‐Aventis Germany Frankfurt am Main Germany; ^3^ Division of Nephrology and Internal Intensive Care Medicine Charité‐Universitätsmedizin Berlin Germany; ^4^ Department of Rheumatology and Clinical Immunology Charité‐Universitätsmedizin Berlin Germany

**Keywords:** bone marrow, cytokines, hematopoietic cells, single cell sequencing, stromal cells

## Abstract

Bone marrow (BM) stromal cells are important in the development and maintenance of cells of the immune system. Using single cell RNA sequencing, we here explore the functional and phenotypic heterogeneity of individual transcriptomes of 1167 murine BM mesenchymal stromal cells. These cells exhibit a tremendous heterogeneity of gene expression, which precludes the identification of defined subpopulations. However, according to the expression of 108 genes involved in the communication of stromal cells with hematopoietic cells, we have identified 14 non‐overlapping subpopulations, with distinct cytokine or chemokine gene expression signatures. With respect to the maintenance of subsets of immune memory cells by stromal cells, we identified distinct subpopulations expressing *Il7*, *Il15* and *Tnfsf13b*. Together, this study provides a comprehensive dissection of the BM stromal heterogeneity at the single cell transcriptome level and provides a basis to understand their lifestyle and their role as organizers of niches for the long‐term maintenance of immune cells.

## Introduction

Bone marrow (BM) stromal cells provide distinct niches for the maintenance and development of hematopoietic cells, including various cells of the immune system 1, 2, 3, 4, 5, but how the diversity of hematopoietic cells is matched by the diversity of mesenchymal stromal cells organizing their niches is poorly understood 6, 7. In vivo, BM stromal cells have been shown to express vascular cell‐adhesion molecule 1 (VCAM1; CD106) 8, CXCL12 and IL7, collagen II and XI 1, 2, 3, 4, 5, PDGFRB (platelet‐derived growth factor receptor B), CDH11 (Cadherin 11), LepR (Leptin receptor), Nestin and other genes 9, 10, but a comprehensive analysis of their individual gene expression profiles has been missing.

In the present study, we describe a novel protocol for the isolation of BM stromal cells ex vivo by fluorescence‐activated cell sorting, yielding more than 95% purity and more than 60% recovery. We have determined and describe here the individual, complete transcriptomes of more than 1000 individual BM stromal cells by single cell RNA sequencing (scRNA‐*seq*). These cells show a remarkable heterogeneity, in particular with respect to the expression of genes encoding cell‐bound and secreted molecules involved in the communication of stromal cells with cells of the hematopoietic system. We have identified distinct stromal subpopulations, which qualify to organize specific niches for distinct immune memory as well as hematopoietic cells.

## Results and discussion

### Isolation of individual BM stromal cells

**Figure 1:**
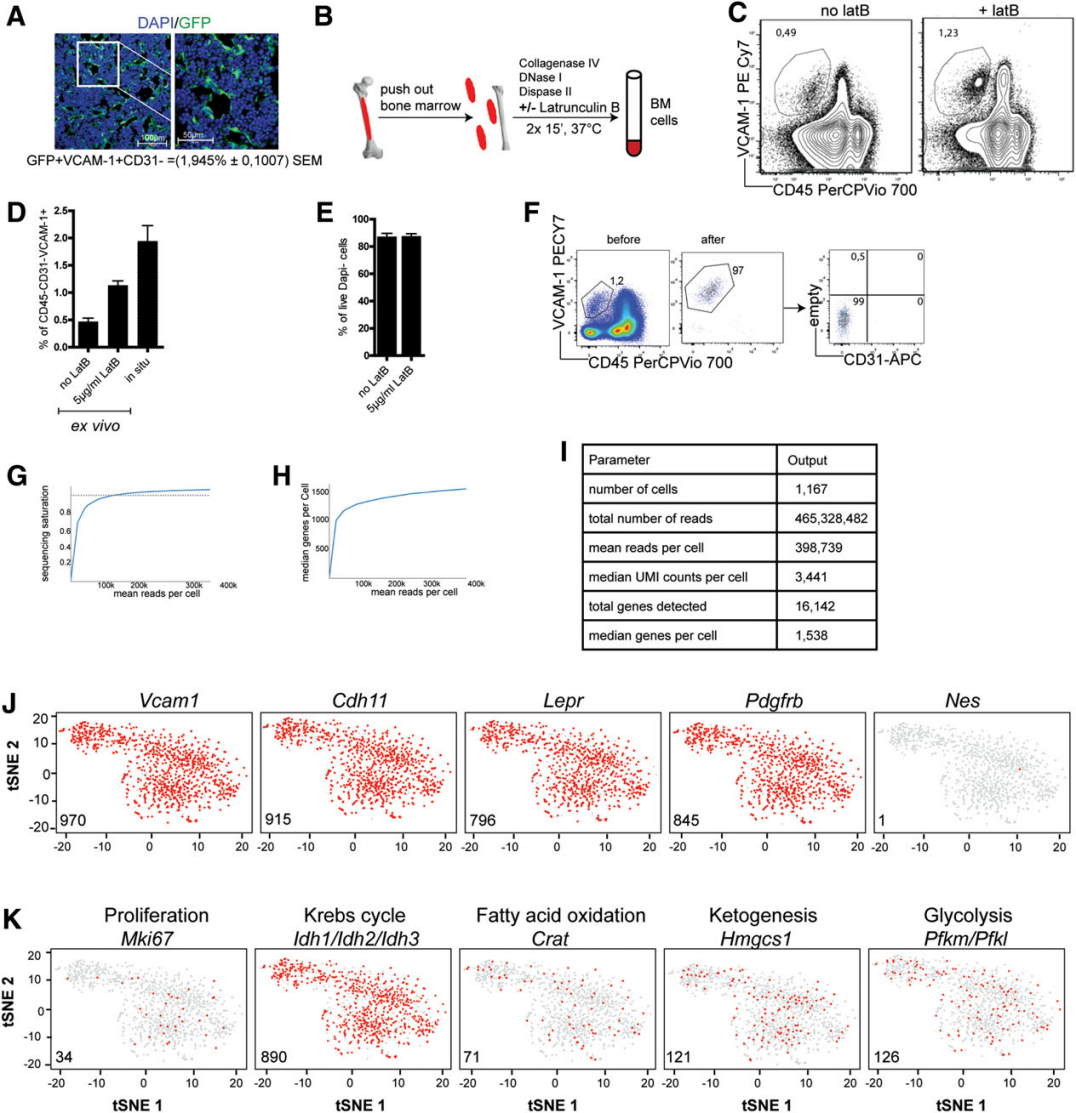
*Isolation and single cell sequencing of* ex vivo *VCAM+CD45‐CD31‐Ter119‐ BM stromal cells*. (A). In situ quantification of BM reticular stromal cells: DAPI+GFP+(VCAM‐1+CD31‐) reticular cells constituted 1.945% ± 0.1007 SEM of BM cells. Representative image of analysis of 30 histology sections from 5 different mice in 3 independent experiments. Scale bars: 100 and 50 µm,20x magnification (B) Schematic overview of isolation of BM stromal cells. (C) Representative dot plots of VCAM‐1 against CD45 gated on CD31‐Ter119‐Dapi‐ comparing isolation with or without Latrunculin B. (D) Frequencies of ex vivo BM VCAM‐1+ stromal cells isolated with or without addition of Latrunculin B compared to those determined in situ. (E) Frequencies of DAPI‐ (live) BM cells isolated with or without addition of Latrunculin B. (F) Representative plot of cytometric sorting of *ex‐vivo* BM VCAM‐1+CD45‐CD31‐Ter119‐ cells (G‐I) Quality assessment of the 10x genomic sequencing, showing sequencing saturation (G) and median genes per cell (H) against the mean reads per cell and the summary of the sequencing (I). (J) t‐SNE plots highlighting the expression (red) of individual BM stromal markers. (K) t‐SNE plots showing the expression (red) of genes associated with cellular function of proliferation (cell cycle) and metabolism in individual cells. Data from (C and E) represent pooled results from 4 independent experiments each with 3–5 mice per group. Data from E is extracted from results of experiments described in (A and C). The t‐SNE analyses shown in Fig. 1J and 1K are based on *n* = 1035 individual stromal cells.

In order to estimate the size of the stromal compartment in the BM, we have determined the frequency of radiation‐resistant reticular cells in Ubiquitin:GFP chimeric mice 1, by fluorescence microscopy. The GFP+VCAM‐1+CD31‐ reticular cell compartment constituted about 2% (1.945% ± 0.1007 SEM) or ∼5 × 10^6^ of all BM cells in situ (Fig. 1A) 2. Since BM stromal cells form a tight reticular network, their isolation as individual cells provides a challenge. Conventional single cell preparation methods use mechanical disruption and enzymes targeting adhesive extracellular matrix (collagenase, DNAse and dispase) 11. To break and prevent re‐adhesion of stromal cells, we here describe the usage of Latrunculin B, a drug interfering with the polymerization of actin 12. Addition of Latrunculin B to the digestion cocktail significantly doubled, as compared to isolation without Latrunculin B (Fig. 1B and1C), the recovery of ex vivo isolated BM stroma cells. This cell recovery is about 60% of the cell numbers estimated in situ (Fig. 1C and1D). More important, the addition of Latrunculin B did not affect the viability of the cells (Fig. 1E). Consequently, isolation of stromal cells was always performed with the addition to Latrunculin B to the digestion cocktail.

### Single cell transcriptomes of BM stromal cells

Ex vivo VCAM‐1+CD45‐Ter119‐CD31‐ BM cells were sorted by FACS to 97% purity (Fig. 1F) and transcriptomes of individual cells were determined using 10X genomics‐based droplet sequencing. Transcriptomes of 1167 individual stromal cells were analyzed with a mean of 398,739 reads per cell resulting in a saturation rate of 95.6% (Fig. 1G), i.e., more than 95% of each transcriptome was captured. A total of 16,142 genes were detected in total, with a median of 1,538 genes per cell (Fig. 1H and 1I). We used the entire transcriptomes of each cell to perform a t‐distributed stochastic neighbor embedding (t‐SNE) analysis 13 and visualize the basic heterogeneity of the cells. Within the t‐SNE plots, genes of interest expressed by cells are highlighted in red.

More than 90% of the BM stromal cells expressed the genes *Vcam1, Pdgfrb, LepR, Cadherin 11*(*Cdh11*), qualifying these genes as genuine stroma cells markers, but also confirming the quality of the cells 14 (Fig. 1J). The stromal cells did not express the pericyte marker nestin (*Nes)*, 15 (Fig. 1J).

Most of the cells were resting in terms of proliferation, since they did not express the proliferation marker *Mki67* 16 (Fig. 1K), confirming earlier results obtained with EdU pulse chase labelling 3. Nearly all cells expressed at least one of the Isocitrate dehydrogenases isoforms (*Igh1, Idh2* or *Idh3)*, the rate limiting enzyme of the TCA 17 (Fig. 1K). With respect to the energy source of metabolism, stromal cells were heterogeneous, some expressing rate limiting enzymes *Pfkm and Pfkl* of the glycolytic pathway 18, or *Crat* for fatty acid oxidation 19 or *Hmgcs1* for ketogenesis 20.

**Figure 2:**
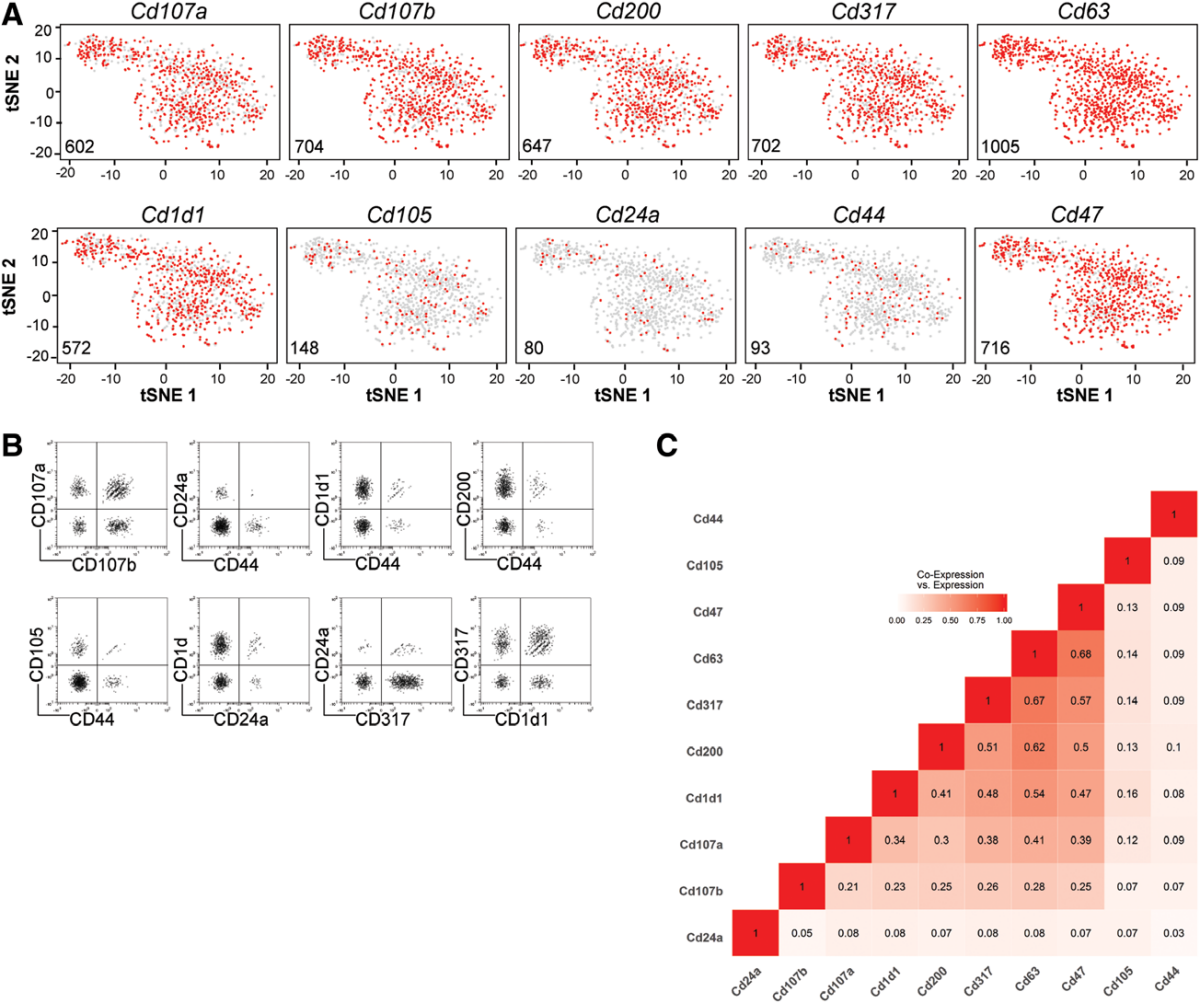
*Expression of genes encoding CD markers*. The experimental procedure is the same as described in the legend of Fig. 1. (A) t‐SNE plots highlighting the distribution and expression (red) of genes encoding for surface markers. (B) Scatterplots; Co‐expression of CD genes as found by normalized unique molecular identifier‐counts (UMI‐counts) from sequencing. Co‐expression of genes were arcsinh‐transformed for flow cytometric‐like visualization, an artificial noise was subtracted to 0 counts .(C) Co‐expression of selected CD‐marker genes as defined by Jaccard similarity coefficient (Proportion of cells expressing two or at least one marker). The t‐SNE analysis shown in Fig. 2 is based on *n* = 1035 individual stromal cells.

Genes encoding cell surface molecules were often expressed individually by the stromal cells, as exemplified here for *Lamp1* (*Cd107a*), *Lamp2* (*Cd107b*), *Ox2* (*Cd200*), *Bst2* (*Cd317*), *Cd1d1*, *Cd63*, *Cd105*, *Cd24a*, *Cd44 and Cd47* (Fig. 2A). At the level of single cell transcriptomes, cells expressing the various cluster of differentiation (CD) genes (Fig. 2A) are dispersed over the t‐SNE plots. This observation suggests that stromal cells expressing or not a respective CD marker are closely related and do not necessarily represent distinct subpopulation. However, subpopulations expressing distinct combinations of CD markers can readily be identified by contrasting their expression as found by sequencing (Fig. 2B) and the proportion of cells expressing two or at least one of the genes encoding for surface proteins (Fig. 2C).

### Cytokine and chemokine expression is restricted to distinct subsets of stromal cells

**Figure 3:**
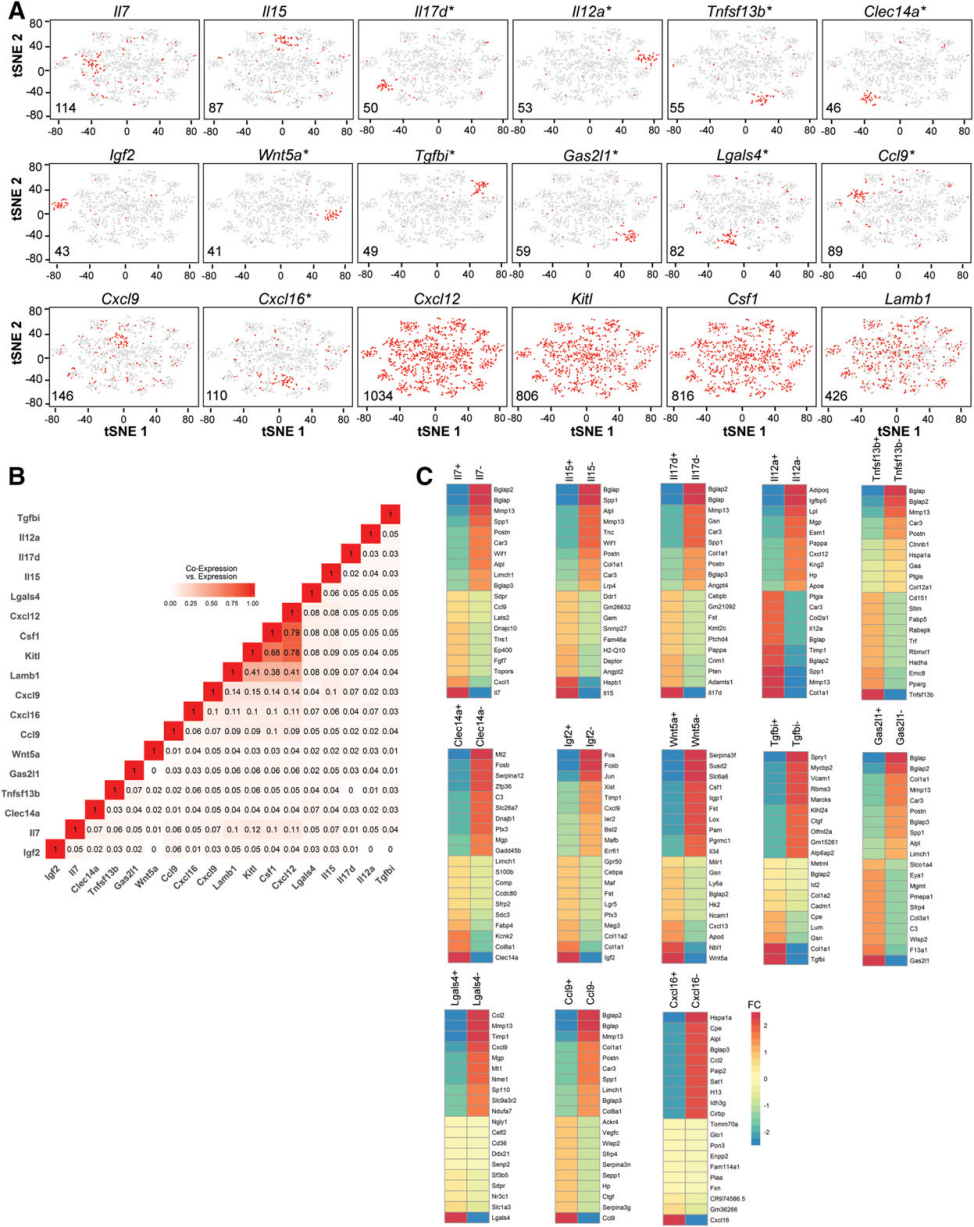
*Cytokine and chemokine expression is restricted to distinct subsets of stromal cells*. The experimental procedure is the same as described in the legend of Fig. 1. (A) t‐SNE plots of supervised clustering of cells using 108 genes encoding secreted factors with known role in communication of stromal cells with cells of the hematopoietic system. Cells expressing a particular gene are highlighted in red (* Defines stable clusters as defined by Consensus Clustering based on random t‐SNEs and/or Consensus Clustering as proposed by Kiselev and colleagues 21). (B) Co‐expression of selected communication genes as defined by Jaccard similarity coefficient (Proportion of cells expressing two or at least one marker). (C) Comparisons of gene expression profiles expressing selected marker genes forming stable clusters. Fold change (FC) shows the log2 (Average Expression of positive cells) ‐ log2 (Average Expression of negative cells), displayed are the top 10 genes with the highest fold change. DiffExpTest‐method was used for the statistical analysis of differential expressed genes. The t‐SNE analysis shown in Fig. 3 is based on *n* = 1035 individual stromal cells.

In the interaction between stromal cells and hematopoietic cells, the expression of chemokines and cytokines by stromal cells is essential for them to attract and control hematopoietic cells. Thus, we analyzed the stromal cell transcriptomes for the expression of genes which encode for secreted proteins. We selected 108 genes (Supporting Information 1A) for further analysis, based on their established role in the communication of stromal cells with cells of the hematopoietic system. 37 of 108 selected genes were differentially regulated and were used for a supervised clustering analysis (Materials & methods section for detailed description). 14 non‐overlapping cytokine/chemokine subsets of stromal cells were identified by the clustering analysis (Fig. 3A). In contrast, genes like *Cxcl12*, *Kitl*, colony stimulating factor 1 (*Csf1*) and Laminin B1 (*Lamb1*), were expressed by most stromal cells, hence they do not define distinct subpopulations of stromal cells based on positive and negative expression (Fig. 3A). Although *Cxcl12* is expressed in almost all stromal cells, we identified three subpopulations of stromal cells according to the expression level. 126 cells (∼12%) expressed low amounts (*Cxcl12lo*; < 4 ln normalized unique molecular identifier (lnUMI) counts per cell; average of 3.06 lnUMI counts), 80 cells (∼8%) with intermediate expression level (*Cxcl12int*; ≥4 and ≤5 ln UMIs; average of 4.59 lnUMIs per cell) and 829 cells (80%) expressing high levels of *Cxcl12* (*Cxcl12high*; >5 lnUMIs; average of 5.74 lnUMIs per cell) (Supporting Information Fig. 2A). The three *Cxcl12* subpopulations differ in their molecular signatures and could potentially have different functions within the bone marrow (Supporting Information Fig. 2B).

In order to test the stability of the identified clusters, we applied Consensus Clustering based on random t‐SNEs as well as Consensus Clustering as described by Kiselev et al. 21. Both methods verified the stability of most of the identified clusters except the cluster for *Cxcl9*. The clusters for *Cxcl16* and *Il15* expressing stromal cells could be verified by the random t‐SNE approach but not by the Consensus Clustering method from Kiselev et al. In addition, we identified clusters of stromal cells expressing *Il4ra and Tgfbr1* by random t‐SNE approach as well as clusters for *Il17rd, Ccl7, Cxcl1 and Cxcl10* by using both stability algorithms (Supporting Information Figs. 2 and 3). Thus, cells expressing the cytokines *Il7, Il15, Il12a, Il17d, Clec14a, Igf2, Lgals4, Tnfsf13b, Il4, Wnt5a and Tgfbi*, or the chemokines *Ccl9, Cxcl16* form unique subsets of bone marrow stromal cells (Fig. 3A).

IL17D is a novel cytokine which inhibits the development of myeloid progenitor cells 22. CLEC14A is a type I transmembrane involved in cell‐to‐cell adhesion, and thus shaping immune response 23. IL12A has multiple effects on T and natural killer cells 24. CXCL16 attracts memory T cells which express CXCR6 25. CCL9 and CCL7 attract subsets of dendritic 26 and monocytes, respectively 27. Expression of any of these chemokine/cytokine genes was indeed exclusive to distinct stromal cells, with less than 10% of cells co‐expressing any two of these genes as estimated by the Jaccard similarity coefficient (Fig. 3B). Furthermore, stromal cells expressing these cytokine and chemokine genes express defined gene signatures, based on their entire transcriptomes, qualifying them as distinct subpopulations of stromal cells (Fig. 3C).

### Concluding remarks

BM has been identified as the residency of immune memory cells providing long‐term protection against systemic pathogens. BM stromal cells have been postulated to organize the survival niches for these memory cells 1, 2, 3, 4, 5. Analyzing the individual transcriptomes of more than 1000 murine BM stromal cells, we find a tremendous heterogeneity, with essentially no two cells expressing the same transcriptome. Nearly all stromal cells express CXCL12, a critical signal to attract immune memory cells or their precursors. Distinct subsets of stromal cells express IL‐7 or IL‐15, cytokines which have been invoked in the maintenance of CD4 and CD8 memory lymphocytes 28, 29. More than 5% of the stromal cells express *Tnfsf13b*, the gene encoding for the protein BAFF (B‐cell activating factor), a cytokine critical for the maintenance of memory plasma cells 30. Thus, BM stromal cells are potentially autonomous in providing niches for the long‐term maintenance of immune memory cells. With regards to the maintenance of hematopoietic stem cells and early progenitors; *Cxcl12* is expressed by all stromal cells whiles *Kitl* and *Csf1* are expressed by about 80% of cells (Fig. 3A). *Flt3l* and *Il7* are expressed by small fraction of BM stromal cells. In perspective, this data set provides a considerably fundus towards an understanding of the interaction of stromal cells and hematopoietic cells on the single cell level.

## Materials and methods

### Mice

IL‐7‐GFP knock‐in mice were kindly provided by Koichi Ikuta (Kyoto University, Japan). C57BL/6J and mice expressing GFP under control of the ubiquitin promoter (Ubq:GFP) were obtained from Jackson Laboratories (Germany) and housed under specific pathogen‐free conditions at the DRFZ, Berlin. All experiments were approved by the federal state institution “Landesamt für Gesundheit and Soziales” (T0192/10),Berlin, Germany.

### Single cell suspension of BM

BM flush‐out and the empty bones (tibia and femur) were digested with 0.5 mg/ml collagenase IV (Sigma‐Aldrich), 1 mg/ml DNase I (Sigma‐Aldrich), 0.25 mg/mL Dispase II (Roche), with or without 5 µg/mL Latrunculin B (Sigma‐Aldrich), for 30 min at 37°C.

### Flow cytometry

Flow cytometry and cell sorting were performed as described 31. The following antibodies were used: anti‐CD45(30F11), anti‐VCAM‐1(429), anti‐CD31(390), anti‐Ter119(Ter119), antibodies were purchased from Miltenyi Biotec, Biolegend, or produced in DRFZ. Dead cells were excluded by DAPI. Flow cytometric data were acquired on MACSQuant (Miltenyi Biotec). BDInflux cell sorter (BD Bioscience) was used for cell sorting. Flow cytometric data were analyzed with FlowJo (Tree Star, Inc.).

### In‐situ quantification of radiation resistant BM stromal cells

Chimeric mice were generated as previously described 1. Briefly, mice that express GFP ubiquitously were irradiated and reconstituted with BM cells from C57BL/6J mice. Immunofluorescence staining of BM sections was performed according to established protocol 1 using the following antibodies: anti‐VCAM‐1(429) and anti‐CD31(390). For the nuclear staining, sections were stained with 1 µg/mL DAPI in PBS. Images were acquired using a Zeiss LSM710 confocal microscope with a 20 × /0.8 numerical aperture objective and were analyzed with Zen 2009 Light Edition software (Carl Zeiss Micro Imaging).

### Single cell RNA‐sequencing

For single cell library preparation, ex vivo FACS sorted VCAM‐1+CD45‐Ter119‐CD31‐ BM cells were applied to the 10X Genomics platform using the Single Cell 3’ Reagent Kit V2 (10x Genomics) following the manufacturer's instructions. Upon adapter ligation and index PCR, the quality of the obtained cDNA library was assessed by Qubit quantification, Bioanalyzer fragment analysis (HS DNA Kit, Agilent) and KAPA library quantification qPCR (Roche). The sequencing was performed on a NextSeq500 device (Illumina) using a High Output v2 Kit (150 cycles) with the recommended sequencing conditions (read1: 26nt, read2: 98nt, index1: 8 nt, index2: n.a.).

### Sc RNA‐seq analysis

Illumina output was demultiplexed and mapped to the mm10 reference genome by cellranger‐2.0.2 (10x Genomics Inc.) using refdata‐cellranger‐mm10‐1.2.0 in default parameter setting and 3000 expected cells. Raw UMI‐counts were further analyzed using R 3.5.2 with Seurat package 32, as proposed by Butler and colleagues 33, including log‐normalization of UMI‐ counts, detection of variable genes and scaling. T‐distributed Stochastic Neighbour Embedding and the underlying Principle Component Analysis was performed based on 30 components using variable genes and a perplexity of 30 as set by default. Potential lymphocyte and erythrocyte contamination cells expressing *Ptprc* (CD45) or hemoglobin subunits (Hba) respectively were detected and excluded. Data were reanalyzed after excluding the contaminates using the remaining 1035 stromal cells (Fig. 1 J‐L and 2A). Scatterplots for co‐expression of genes were based on normalized UMI‐counts, with an artificial noise subtracted form 0 counts for visualization (Fig. 2B). Co‐expression matrices were based on the Jaccard similarity coefficient of cells expressing two or at least one gene (Fig. 2C and Fig. 3B). Heat maps (Fig. 3C) show the log‐transformed fold change of mean expression of positive and negative cells, displayed are the top 10 genes with the highest fold change. DiffExpTest‐method was used for the statistical analysis of differential expressed genes 34. The single cell RNA sequencing data reported in this paper have been deposited in the Gene Expression Omnibus (GEO) database, https://www.ncbi.nlm.nih.gov/geo (accession no. GSE131365).

### Analysis of stromal communication genes

For the analysis of stromal communication genes, a set of 108 genes were derived from literature (Supporting Information 1A). Out of these, 37 were detected as variable and used for t‐SNE (Fig. 3A). Cluster stability was analyzed using random t‐SNEs as well as Consensus Clustering as described by Kiselev and colleagues 21. 1000 random t‐SNEs were generated based on 80% of cells, using random seeds for both t‐SNE and cell sampling. Clusters within each t‐SNE were determined by density‐based clustering (DBSCAN) as implemented in the java Apache Commons Mathematics Library “common.math3‐3.4.1”, using Euclidian‐Distance, minimum number of 10 cells for a cluster and an average distance to the tenth’ neighbor as the Epsilon‐neighburhood. The consensus was defined as the ratio of co‐occurrence of two cells in the same cluster and same random t‐SNEs. Hierarchical clustering of cells was performed based on complete linkage and Euclidian Distance. Main clusters were defined by cutting the tree at 95% of its height, leading to 24 Clusters with more than 10 cells (Supporting Information 3A). Cluster stability is visualized by Silhouette‐Plot (Supporting Information 3B). Markers for clusters were determined by the area under the receiver operating curve (AUC) based on the expression of the respective gene. Markers were defined by a threshold of AUC > = 0.95 (Supporting Information Fig. 3C). All markers were statistically significant with p‐values < = 2.E‐8 as determined by the Mann–Whitney‐U‐Test. The Consensus Clustering was performed for 2 to 50 expected clusters in default settings but disabling gene‐filtering 21. The optimal number of clusters was defined by the highest mean average silhouette width discarding clustering 2, 3 and 4 after visual inspection of the consensus matrix (Supporting Information Fig. 4A‐C). Markers for the Consensus‐Clusters were defined by AUC > = 0.95.

## Conflict of interest

The authors declare no financial or commercial conflict of interest.

**Abbreviations**
BM: Bone marrow
CD: Cluster of differentiation

## Supporting information

Supporting Information Information
