# Supplementary material for: Single‐cell transcriptomes of murine bone marrow stromal cells reveal niche‐associated heterogeneity
Source: Eur J Immunol. 2019 Jun 7;49(9):1372–9. doi: 10.1002/eji.201848053 (PMC6771914; doi:10.1002/eji.201848053)
Supplement: Supplementary file 1 — Supporting Information Information [file EJI-49-1372-s001.pdf]

European Journal of Immunology

Supporting Information
for
DOI 10.1002/eji.201848053

Richard K. Addo, Frederik Heinrich, Gitta Anne Heinz, Daniel Schulz, Özen Sercan-Alp, Katrin Lehmann, Cam Loan Tran, Markus Bardua, Mareen Matz, Max Löhning, Anja E. Hauser, Andrey Kruglov, Hyun-Dong Chang, Pawel Durek, Andreas Radbruch and Mir-Farzin Mashreghi

Single-cell transcriptomes of murine bone marrow stromal cells reveal niche-associated heterogeneity

© 2019 The Authors. *European Journal of Immunology* published by WILEY-VCH Verlag GmbH & Co. KGaA, Weinheim.

Supplemental Figure 1

A

Gene	Number of cells
Adipoq	901
Alpl	380
Angpt1	216
Angpt2	206
Angpt4	266
Angptl1	226
Angptl2	51
Angptl4	532
Apln	4
Bgn	1026
Ccl2	119
Ccl25	24
Ccl7	48
Ccl9	89
Clec11a	547
Clec14a	46
Clec16a	33
Clec2d	437
Clu	900
Colec12	634
Cp	945
Csf1	816
Ctgf	349
Cx3cl1	229
Cxcl1	66
Cxcl10	35
Cxcl12	1034
Cxcl13	86
Cxcl14	955
Cxcl16	110
Cxcl2	26
Cxcl5	26
Cxcl9	146
Cxcr2	18
Cyr61	569
Fst	472
Fstl1	873
Fstl3	22
Gas1	213
Gas2l1	59
Gas6	1006
Gas7	68
Gas8	39
Gdf10	172
Gdf11	37
Grn	608
Gsn	441
Hp	996
Igf1	514
Igf2	43
Il10rb	338
Il11ra1	195
Il12a	53

Gene	Number of cells
Il12rb1	26
Il15	87
Il15ra	25
Il17d	50
Il17ra	213
Il17rc	142
Il17rd	84
Il18	21
Il18bp	76
Il1r1	264
Il1rl1	1
Il1rn	411
Il2	131
Il20ra	15
Il20rb	13
Il34	515
Il3ra	215
Il4ra	110
Il6st	430
Il7	114
Kitl	806
Kng1	633
Kng2	683
Lgals1	777
Lgals3	21
Lgals3bp	607
Lgals4	82
Lgals8	190
Lgals9	159
Lgalsl	91
Lpl	896
Mdk	802
Ngp	483
Ogn	554
Rarres2	906
Sfrp1	251
Sfrp2	146
Sfrp4	753
Spp1	1020
Tgfb1	164
Tgfb2	55
Tgfb3	164
Tgfbi	49
Tgfbr1	107
Tgfbr2	132
Tgfbr3	594
Tnc	949
Tnfrsf12a	30
Tnfrsf19	486
Tnfrsf1a	593
Tnfrsf1b	151
Tnfrsf21	197
Tnfsf11	171

Gene	Number of cells
Tnfsf12	324
Tnfsf13b	55
Wnt4	141
Wnt5a	41
Wnt5b	49

B

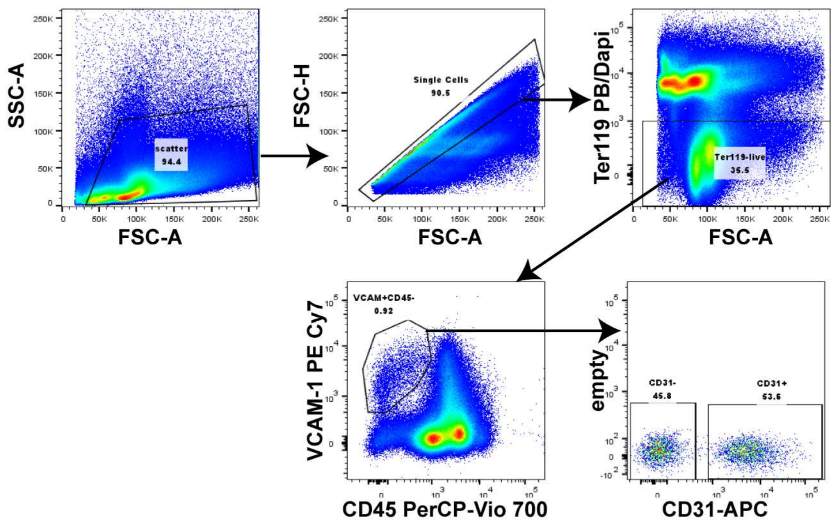

Supplemental Figure 2

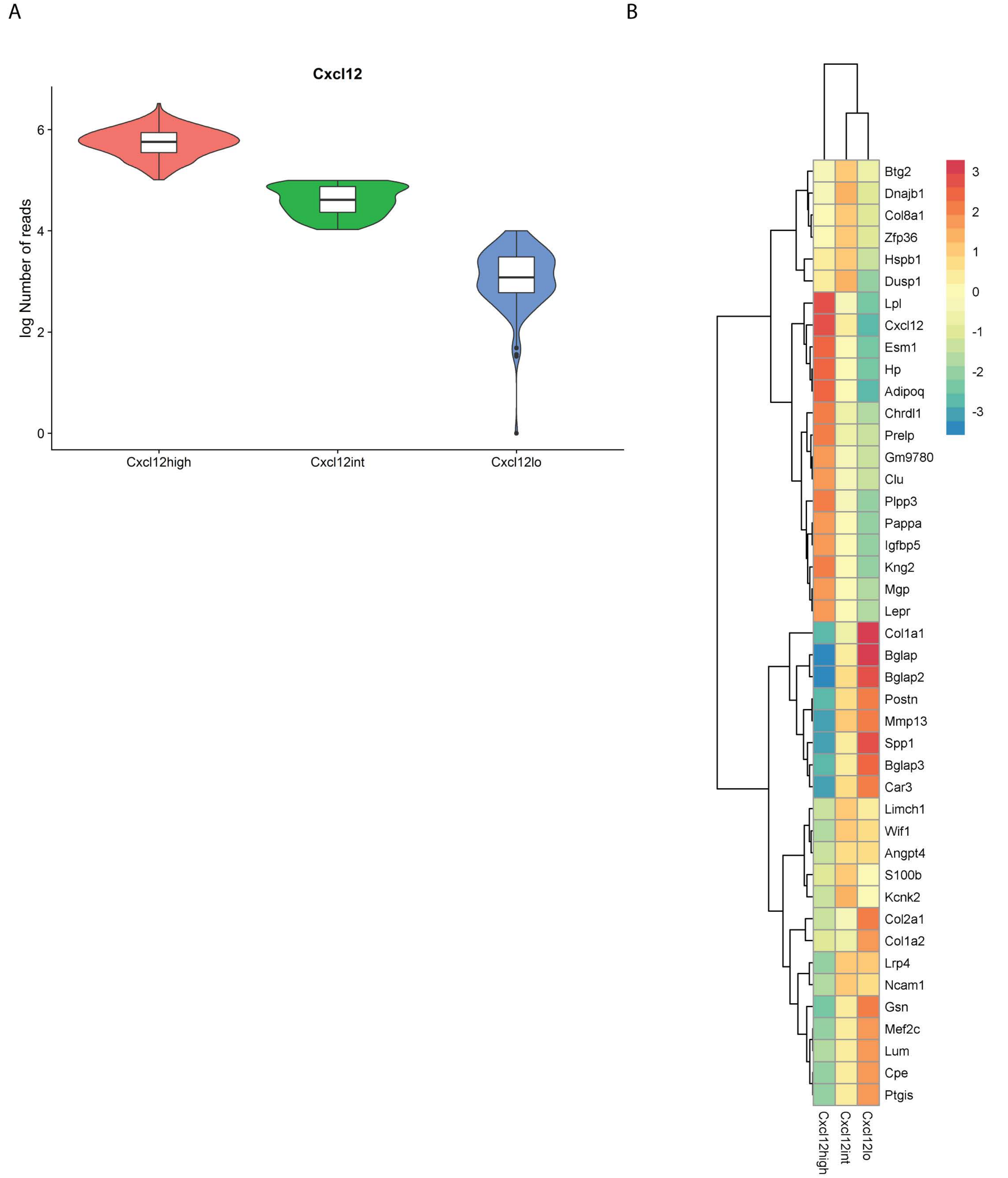

Supplemental Figure 3

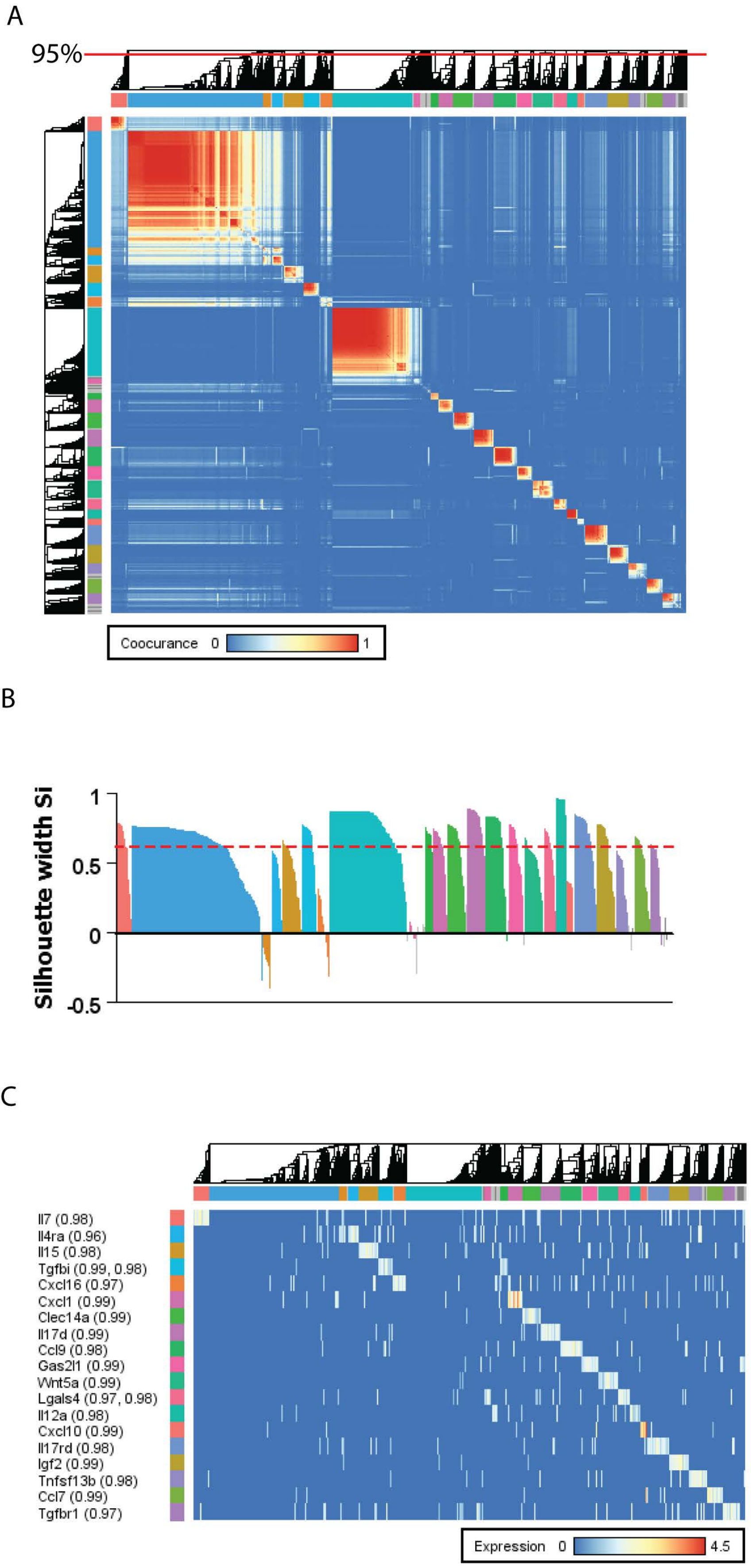

Supplemental Figure 4

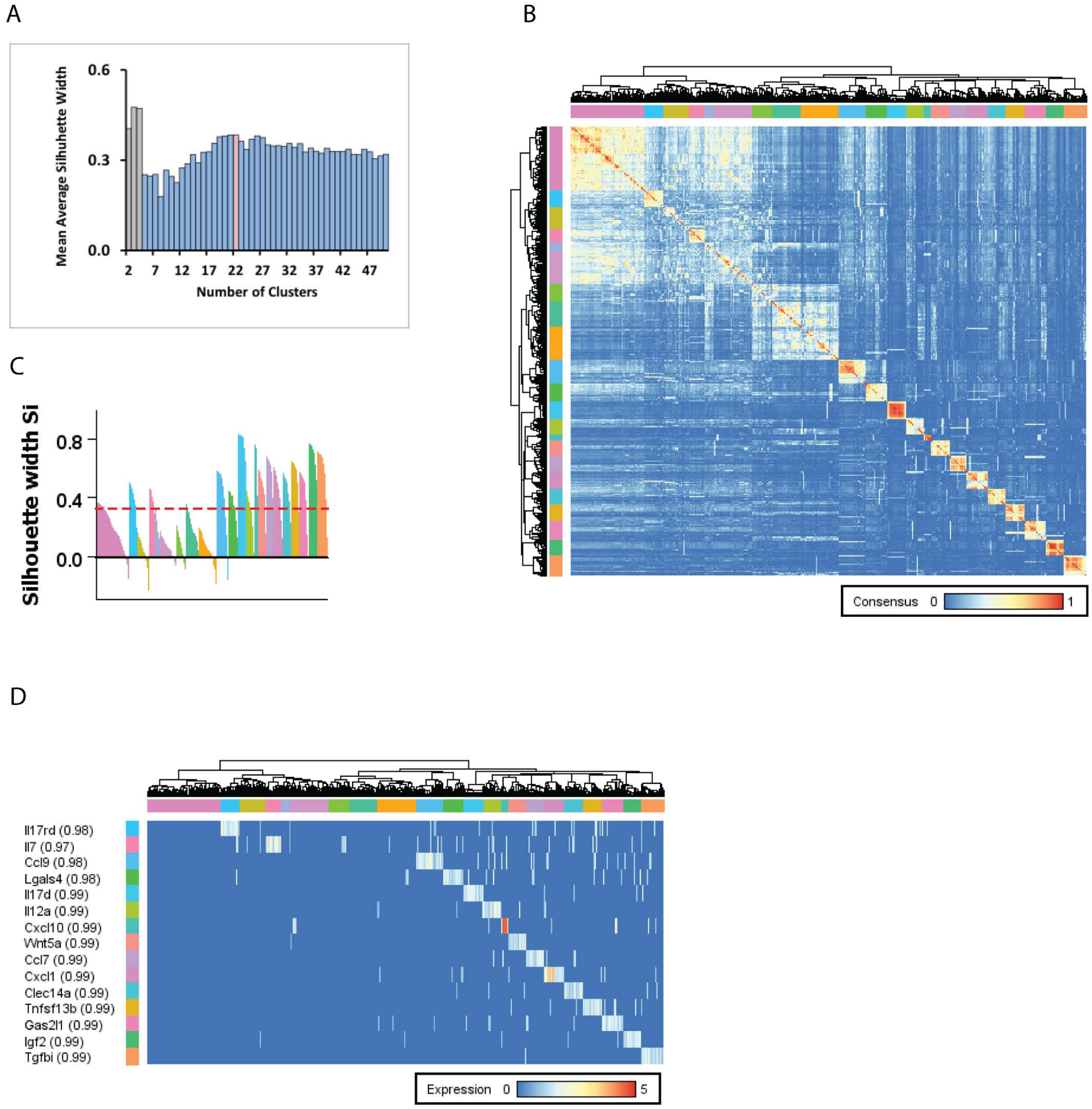

Supplementary Figure 1

(A) Selected 108 genes encoding for factors with established role in communication of stromal cells with cells of the hematopoietic system. (B) Gating strategy for the flow cytometric identification of murine BM VCAM-1+ stromal cells .

Supplementary Figure 2

Violin plot showing stromal cell populations expressing different levels of Cxcl12.

(A) 126 cells expressed low amounts (*Cxcl12lo; mean ln of normalized UMI counts of* 3.06 per cell)–80 cells showed intermediate expression level (*Cxcl12int*; mean *ln of normalized UMI counts of* 4.59 per cell)) and 829 cells expressed high levels of *Cxcl12* (Cxcl12high; mean *ln of normalized UMI counts of* 5.74 per cell). (B) Expression levels of the top 15 differentially expressed genes in the three *Cxcl12* populations, as defined by the fold change between each subset to the rest. Solely genes with a $p < 0.05$ and log2 fold change > 1.3 were considered.

Supplementary Figure 3

Density-based analysis of cluster stability for 37 stromal communication genes.

A) Consensus matrix of co-occurrence of cells in 1000 TSNE plots based on random seed for TSNE and 80% sampled Cells communication genes. Cells were clustered based on percentage of co-occurrence in same cluster, complete linkage and Euclidian Distance. Clusters were defined by cutting the hierarchical tree at 90% of high (red line). Clusters comprising less than 10 cells are colored in gray shades. B) Silhouette-Plot of clusters, with the average silhouette width of 0.56 denoted in dashed red line. C) Marker genes for cluster, with AUC-Values denoted in brackets and expression as defined by ln of normalized UMI counts.

Supplementary Figure 4

Consensus Clustering of 37 stromal communication genes.

A) Mean Average Silhouette width's (ASW) for different number of expected clusters k.

Clustering with the highest Mean ASW is colored in red. Clustering for k 2-4 were not considered after visual inspection of the respective consensus heat maps (gray color). B) Consensus heat map for clustering with k of 22. C) Silhouette-Plot for clustering with k of 22, with the average silhouette width of 0.33 denoted in dashed red line. D) Marker genes for cluster, with AUC-Values denoted in brackets and expression as defined by ln of normalized UMI counts. Only markers with AUC > 0.95 are shown.
